# Focus on the endocrine system of children born after reproductive technologies in Kazakhstan

**DOI:** 10.5339/qmj.2025.9

**Published:** 2025-02-23

**Authors:** Sevara Ilmuratova, Lyazzat Manzhuova, Aigul Bazarbayeva, Vyacheslav Lokshin, Zhanar Nurgaliyeva, Farida Kussainova

**Affiliations:** ^1^Department of Science and Consulting, Kazakhstan Medical University KSPH, Almaty, Republic of Kazakhstan; ^2^Department of Science and Postgraduate Education, Scientific Center of Pediatrics and Children's Surgery, Almaty, Republic of Kazakhstan; ^3^Department of Assisted Reproductive Technologies, International Clinical Center for Reproductology PERSONA, Almaty, Republic of Kazakhstan; ^4^Department of Childhood Diseases named after Professor N.A. Barlybaeva, Asfendiyarov Kazakh National Medical University, Almaty, Republic of Kazakhstan; ^5^Department of Obstetrics and Gynecology, Asfendiyarov Kazakh National Medical University, Almaty, Republic of Kazakhstan *Correspondence: Aigul Bazarbayeva. Email: aigu.bazarbayeva@gmail.com

**Keywords:** In vitro fertilization, assisted reproductive technologies, thyroid-stimulating hormone, somatotropin, offspring, children's health

## Abstract

**Introduction:**

Reproductive technologies are used more widely today than ever before. This increase in the use of assisted reproductive technology (ART) is directly related to sociodemographic conditions that result in delayed childbirth among age groups with lower fertility. Infertility affects 17% of married couples, and in some countries 6% of children are born with in vitro fertilization (IVF). In this context, the aspect of the influence of reproductive technologies on hormonal indicators of offspring in relation to anthropometric data remains insufficiently examined. The purpose of this cohort study is to compare the hormonal panel and anthropometric data of ART-conceived children with the corresponding data of children conceived naturally.

**Methodology:**

Biochemical tests are used to determine the amount of free triiodothyronine (T3) and total thyroxine (T4), somatotropin, insulin, insulin-like growth factor, glucose, potassium, and sodium cations in blood samples from the experimental and control groups.

**Results:**

The results indicate that the use of assisted reproductive technologies neither altered the endocrine panel of the thyroid gland, nor affected other biochemical parameters. Variations in technologies – classical IVF, fresh or frozen embryo transfer, intracytoplasmic sperm injection – also did not affect the quantitative value of the above indicators. Artificial insemination also had no effect on puberty (in both boys and girls). Children born naturally had a greater body weight (3,453 vs 3,160 g, *p* < 0.001) and height (53 vs 51 cm, *p* = 0.002). ART children had significantly higher median free tbl3 levels (3.65 vs 3.48 mU/L, *p* = 0.002) and potassium levels (4.8 vs 4.7 mmol/L, *p* = 0.013), although within the reference ranges. Glucose levels were also higher in ART children (median 4.45 vs 4.29 mg/dl, *p* = 0.01).

**Conclusion:**

Several relationships between biochemical and anthropometric indicators were identified: the correlation between body weight and blood levels of insulin-like growth factor was statistically significant, positive, and weak. The tbl3 level in the experimental group was found to be statistically significant and directly proportional to body height, and insulin content was inversely proportional to body weight. The data obtained make it possible to verify the safety of using a different range of reproductive technologies.

## Introduction

In recent decades, in vitro fertilization (IVF) has become an increasingly common method of conception worldwide due to its effectiveness in overcoming the problem of infertility.^1^ The spread of assisted reproductive technology (ART) is attributed by researchers to sociodemographic factors associated with delayed childbirth in an age group with a lower fertility percentage.^2^ Infertility affects 17% of married couples, and in some countries 6% of children are born with IVF (about 10 million children in total).^3,4^ The spread of reproduction in Kazakhstan began in the middle of the 20th century. In October 1995, the country's first ART laboratory was opened, and in July 1996, a child with IVF was born for the first time in Kazakhstan. The use of ART in the country is developing rapidly, and advanced methods and technologies are being actively introduced, as a result of which more than 30,000 children have already been born.^5^

Most publications in the world are focused on the short-term outcomes of children born after infertility treatment, such as birth weight, multiple births, stillbirths, and live births. However, the focus of researchers’ and clinicians’ attention is now on examining the effect of ART on all areas of child health, including the endocrine status of these children.^6^

Lokshin and Ilmuratova in their study examined the thyroid hormone profile in children conceived via assisted reproductive technologies (aged 4–14 years) and found significantly higher concentrations of thyroid-stimulating hormone (TSH) in these children compared to naturally conceived (NC) children, although the levels remained within the reference range.^6^ Additionally, the study observed more frequent cases of subclinical hypothyroidism and euthyroid hyperthyroidism in the “in vitro” group, regardless of factors such as birth weight, gestational age, size for gestational age, breastfeeding duration, sex, age, body mass index (BMI), thyroiditis, PCOS (polycystic ovary syndrome), diabetes, parity, maternal hypertension, or smoking. The reason for this difference was not an autoimmune factor since thyroid autoantibodies were not detected in children over six months of age. In addition, study participants did not have congenital hypothyroidism or maternal thyroiditis. Lv et al. found that in individuals aged 3–10 years born after fresh embryo transfer (Fresh-ET), the levels of total thyroxine (T4) and TSH were significantly higher and the level of triiodothyronine (T3) tended to increase compared with the group of NC children.^7^ Such data explain the relevance of continuous screening of the endocrine status of children conceived by the IVF method.

The use of ART involves the administration of sex hormones to a woman during the preparation for the program itself and during controlled ovulation stimulation. Even physiological humoral disorders during gestation can cause an inflammatory process, as well as hormonal and metabolic transformations. Conflicting data have been published on the effect of endocrine therapy in IVF programs on the development of inflammation and diabetes due to the risk of pregnancy complications.^8^ There is also evidence that an environment with a high estradiol content in mothers in the first trimester of ART pregnancy correlates with the possibility of thyroid dysfunction in their offspring.^7^

In experiments with laboratory mice, IVF is associated with glucose intolerance.^9^ However, the relationship between glucose metabolism and insulin balance in the first months of pregnancy in humans has not yet been sufficiently investigated. To date, there is evidence that, unlike natural pregnancy, there is an increased risk of GDM (gestational diabetes mellitus) in IVF cases, which depends on a woman's age and increased BMI.^10–12^ The IVF process carries the risk of obesity.^11^ Further studies on the aspect of stimulating the development of diabetes due to the use of endocrine therapy in IVF are needed. They will help identify the potential causes of diabetogenic alertness in offspring.

The likelihood of consequences for metabolic pathologies in children conceived through artificial insemination has attracted particular interest over the past decade and to date has had conflicting data. Fauser et al.^13^ proved that children conceived with ART suffer from hyperglycemia and hypertension, are underweight at birth, and have a greater amount of subcutaneous fat. In addition, it is known that insulin sensitivity is lower in the group of ART children in contrast to NC children. Therefore, potential early metabolic disorders may become more complicated with age and cause chronic pathologies.^14–16^ In a systematic review, Guo et al. found that fasting insulin levels were significantly higher in children with IVF and/or intracytoplasmic sperm injection (ICSI), while fasting glucose levels and the HOMA-IR (homeostasis model assessment of insulin resistance) were comparable to the control sample.^14^ Norrman et al. concluded that ART children were not at risk of developing type 2 diabetes after adjusting for measured intervening factors, but at the same time, they were prone to an increased BMI compared with NC children.^17^ Yeung et al. also found no increased cardiometabolic risk in ART children aged 9 years.^18^ Consequently, cardiometabolic outcomes in children born after ART are generally encouraging. However, further research is needed.

Steiner et al. showed that children from single pregnancies conceived after infertility treatment were not exposed to the potential for hormonal disorders.^19^ Investigation of aspects of hormonal homeostasis in children born to couples with an endocrine cause of infertility remains limited. Nevertheless, the results of recently completed studies on the health of young individuals born “from a lab” show that the effects of ART are not as harmful as expected.^20^ The study by Juonala et al., devoted to the examination of pathologies of the heart, blood vessels, and general metabolism, which included men and women aged 22–35 years, did not reveal any differences compared to the control group in terms of potentially dangerous factors (including in terms of markers of atherosclerosis).^21^ A recent prospective experiment on the effects of ART in young people found low levels of subcutaneous and visceral fat. The Growing Up Healthy Study (GUHS) did not confirm data on changes in thyroid hormone synthesis between the main and control samples. ART did not correlate with the development of autoimmune thyroiditis. In addition, no differences could be demonstrated between the transfer groups of fresh and cryopreserved embryos.^22^

Therefore, the aim of this study was to examine the state of the endocrine status of children born after ART.

## Subjects And Methods

This is a comparative cohort study. The number of subjects required to compare the amount of TSH in the peripheral blood of children, depending on the method of conception, was determined according to Lehr's calculations (with a significance level of 0.01). Substituting into the formula the value of the minimum clinically significant difference in TSH content, determined according to the data of a pilot study by the authors involving 20 children, equal to 0.05, the value of the standard deviation equal to 0.116, the minimum volume of each of the compared aggregates was calculated. It comprised 114 patients. The main group of this cohort study based on clinic registers included 120 subjects under 5 years old born with ART, and the control group included 132 NC subjects of the same age. Among the examined individuals, 56.7% (*n* = 68) of the main sample were boys and 43.3% (*n* = 52) were girls, and as for the sample of natural conception, it was 56.8% (*n* = 75) and 43.2% (*n* = 57), respectively. The clinical registers were used to record patient data.

The selection conditions for the study cohort were a successful ART program after IVF and ICSI and the implantation of Fresh-ET or frozen embryo transfer (FET) with the onset of single or multiple pregnancies and childbirth in 2018–2023. The study did not include intrauterine insemination using sperm from a woman's partner or donor, as well as programs with donor gametes and surrogacy. The control group included participants who were younger than 5 years old from spontaneous pregnancy. In both groups, the anamnesis of children and their mothers was collected, and an objective examination (anthropometric indicators and assessment of sexual development), a comparative analysis of two groups of children with the consultation of a pediatric endocrinologist, and laboratory diagnostics of the endocrine status were conducted: thyroid profile (TSH level, free tbl4, free tbl3); regulation of carbohydrate metabolism (levels of insulin-like growth factor 1 (IGF-1), insulin, somatotropin (ST), glucose); indirect determination of the electrolyte state of the adrenal glands (levels of potassium (K) and sodium (Na)) (Table 1). Insulin plays a crucial role in controlling blood sugar levels and is an important marker for assessing metabolic health. Since previous studies have suggested a potential link between ART and metabolic disorders, such as altered glucose metabolism or an increased risk of insulin resistance, measuring insulin levels helps determine whether ART affects children's ability to regulate glucose.

Data were processed using IBM SPSS Statistical 26 software. The Kolmogorov–Smirnov criterion was used to determine the normal distribution in the sample. Multipole conjugacy tables using Pearson and Fisher criteria were applied to assess the intergroup differences in the feature values. The relationships between the variables were evaluated using the odds ratio (OR) indicator. If the frequency is equal to 0 in one of the cases, the Haldane–Anscombe correction is introduced in the calculation of OR. Calculations were carried out using an online calculator. The difference between the samples was considered significant if the significance index did not exceed 0.05. The study project was in accordance with ethical principles and passed the local ethical commission of the Scientific Centre of Pediatrics and Children's Surgery dated April 13, 2022 (protocol no. 2) on the condition that adults consented to the participation of children in the experiment. Informed consent was obtained from parents before the start of the study.

Other potential confounding variables were not fully accounted for, such as family medical history, personal medical history, and the presence of comorbidities. To strengthen the validity of the findings, future studies should include more comprehensive data collection, such as parental medical history, including conditions such as diabetes, thyroid disease, and cardiovascular problems, as well as anthropometric measurements of both parents (height and weight). In addition to these variables, it is crucial to assess children's health status more comprehensively, including any comorbidities or underlying health conditions.

## Results

When examining the data of the mothers of the two study groups, the indicators of obstetric, gynecological, somatic, and endocrine anamnesis were evaluated (Table 2).

Comparing the frequency of multiple pregnancies depending on the method of conception, the difference was significant (*p* < 0.001). The probability of multiple births was 9.65 times higher in the sample of women who resorted to ART than in the sample of natural conception (95% confidence interval (CI): 2.81–33.18). The association between multiple pregnancies and ART was average (*V* = 0.268). The group in which children were conceived naturally had a higher rate of non-developing pregnancies (*p* = 0.037). Women who had conceived children through ART were 2.13 times less likely to have a history of non-developing pregnancy than women whose pregnancy occurred naturally (95% CI: 0.23–0.97). The relationship between the values was weak (*V* = 0.131). There were no statistically significant differences when comparing the frequency of other maternal anamnesis data from both groups.

The data presented in Table 3 were obtained from the authors’ study and the comparison of the anthropometric parameters of the examined children of both groups. In NC children, weight and height measurements at birth and during the examination were found to be significantly higher than those in the ART group, with significant differences indicated by *p* values (*p* < 0.001, *p* = 0.002 for birth measurements and *p* < 0.001 for both height and weight at the time of examination). The body weight of ART children was significantly lower than that of NC children (the medians were 3,160 and 3,453 g). In this case, attention should be paid to the gestational age, which was statistically significantly higher in the NC group (*p* < 0.001). The gestational age of spontaneously conceived children was higher than that of ART-conceived children (medians 39 and 38 weeks, respectively).

The weight-for-age ratio at the time of examination was estimated according to the WHO Child Growth Standards^23^ (Table 4). This ratio did not show any significance regardless of whether the children were conceived naturally or using ART. The WHO Child Growth Standards were also used to assess the height-for-age ratio in children at the time of examination (Table 5).^23^ A comparative assessment of this ratio among children depending on the method of conception had no statistically significant differences. Table 6 presents data on the assessment of weight to height in children in comparison groups. There were no statistically significant differences in weight-to-height ratios depending on whether children were conceived naturally or using ART.

This study also compared the sexual development of boys depending on the conception option ((Table 7). No significant difference was found between the indicators of the assessment of puberty in boys conceived naturally or using ART. The authors compared the frequency of pathologies from the genital organs in boys, taking into account the ART method used (Table 8). Table 8 focuses specifically on the sexual development of boys and reports the number of cases of normal development, unilateral cryptorchidism, and penile hypoplasia. The rest of the participants for whom these specific conditions were not listed were accounted for in the overall cohort but did not present with any notable pathologies related to sexual development. Therefore, they were classified as having normal sexual development, and no other abnormalities were detected in the remaining participants. After comparing the number of pathologies of sexual development in boys, depending on whether the children were conceived using classical IVF or ICSI, no significant difference was found. A comparison of the girls’ sexual development was also conducted depending on the conception option (Table 9).

The difference in thelarche frequency depending on whether the children were conceived naturally or using ART was not statistically significant (*p* = 0.665). However, there was a clear trend towards a higher incidence of thelarche in girls conceived using ART. The volume of laboratory studies on the endocrine status of children was assessed on the basis of data from world studies on the possible effect of drugs used during pre-pregnancy preparation and during pregnancy in women who resorted to the use of ART on the development of gestational diabetes, and established data from a retrospective analysis of women's medical records included in the current scientific and technical program.^11^ A comparative assessment of the endocrine system of ART-conceived or NC children is presented in Table 10.

The levels of free tbl3, glucose, and potassium were significantly high in the main sample (*p* = 0.002, *p* = 0.01, *p* = 0.013, respectively), but the median values fit into the reference values. The levels of other hormones examined did not show a statistical difference between participants conceived through ART and spontaneously conceived children. Additionally, samples for a number of hormones were selected for the next experiment to screen the endocrine status of children born with ART, depending on the method (ICSI or classical IVF) (Table 11). As shown in Table 11, there were no significant differences in endocrine status between children conceived with classical IVF or ICSI. The hormonal status of participants born after the transfer of fresh or frozen embryos was also analyzed (Table 12).

According to the results, the difference in endocrine status between children born after the transfer of frozen or fresh embryos was not significant. The indicators of the percentage ratio of detected abnormalities in the endocrine status of children, depending on the method of conception, are given in Table 13.

When comparing groups of children by height, depending on the presence of deviations in the analyses of children conceived using ART, the following data were obtained (Table 14).

An analysis of the increased amount of free tbl3 in the blood, depending on the growth indicators of children conceived with ART, showed a significant difference (*p* = 0.01). The results were explained by the more frequent detection of elevated free tbl3 levels in the blood among taller children during the examination compared with children of normal height (*p* = 0.016) (*V* = 0.269 – average relationship). Depending on the detection of reduced plasma insulin concentration in ART children, the following results were obtained for the weight groups (Table 15).

A comparison of reduced blood insulin levels across weight groups revealed a significant difference (*p* = 0.001). Specifically, all children in the underweight group (below the 3rd percentile) had reduced insulin levels, compared to 59.6% in the normal weight group (3rd to 97th percentile) and only 20% in the overweight group (above the 97th percentile). This indicates a clear trend that lower insulin levels are more prevalent in underweight children. The results were due to a higher frequency of reduced blood insulin levels in children with normal weight during examination compared with overweight children (*p* = 0.016) (*V* = 0.285 – the average relationship). When comparing the amount of TSH in plasma and growth at the time of examination, the correlation was not statistically significant (*p* = 0.844). A weak correlation was found between the amount of tbl4 in plasma and height at the time of examination (*p* = 0.19; *p* = 0.004). The correlation between plasma tbl3 concentration and height at the time of examination, calculated based on Spearman's rank correlation coefficient, was statistically significant, inverse, and showed weak crowding on the Cheddock scale (*p* = -0.225; *p* = 0.001).

A statistically significant inverse correlation was found between the concentration of somatotropic hormone (STH) and the amount of insulin in the blood of children (*p* = -0.16; *p* = 0.02). When comparing the concentration of IGF-1 in the blood and the weight of children at the time of examination, a statistically significant, positive, and weak correlation was found on the Cheddock scale (*p* = 0.208; *p* = 0.003) between the children's height at the time of examination (*p* = 0.182; *p* = 0.011), blood glucose levels (*p* = 0.153; *p* = 0.028), and blood insulin levels (*p* = 0.219; *p* = 0.002). A statistically significant direct correlation of noticeable crowding was found between insulin and glucose levels in the blood of children born with ART, which was estimated using Spearman's rank correlation coefficient (*p* = 0.504; *p* < 0.001).

## Discussion

When analyzing the anamnestic data of mothers, the high frequency of multiple pregnancies in the cohort of children born with ART and cases of undeveloped pregnancy in the sample of naturally born participants were statistically significant, which confirm the data of previous studies. An interesting fact is that statistically insignificant indicators of gynecological history, such as endometriosis, uterine fibroids, and ovarian cysts, were recorded more often in mothers from the control sample, as well as the frequency of proliferative changes in pelvic organs, such as hyperplasia and uterine polyps, in the ART group. Among mothers who conceived children naturally, the endocrine status was dominated by pathologies of the pituitary gland (prolactinomas) and the thyroid gland with hyperthyroidism. However, the same frequency of hypothyroid thyroid conditions was recorded in both comparison groups. The number of overweight and obese participants was similar in both samples. Interestingly, when analyzing gestational disorders in the endocrine status of mothers during pregnancies conceived with ART, the incidence of gestational diabetes and gestational thyrotoxicosis was higher, which is consistent with previously published data.^10,11^

In this study, the statistical importance of the difference in height and weight immediately after birth in the comparison samples depended on gestational age. When evaluating the median anthropometric indicators in NC children, only the growth rates differed significantly, which was probably due to constitutional characteristics that were not considered in this study. When assessing the height of the subjects relative to age and gender, the prevalence of taller individuals was 4.9% higher in participants born after ART, which was also reported in previous studies.^24–26^ However, the data were not statistically significant. An individual analysis of a sample of tall children revealed no significant abnormalities in the profile of growth hormone and insulin. However, tbl3 levels were 10-fold higher in the group of children born with ART, which requires further investigation. The frequency of children with smaller height was not statistically significant in both groups and ranged from 3 to 5%.

The results of prospective studies indicate the prevalence of obesity in the cohort of children conceived with ART.^17,27^ In this study, when weight by age was assessed individually, the incidence of obesity was found to be two times more common in children born with ART and prevalent among boys. However, no connection with gestational diabetes was identified, which does not exclude the alimentary nature of this condition and a constitutional predisposition. Mothers of obese children also had a high BMI, which was characteristic of obesity and significant in association with the development of obesity in the offspring.^28,29^ The anamnesis revealed that 70% of children had pathological hyperbilirubinemia in the newborn period, 20% had neonatal hypoglycemia on the background of late prematurity. When objectively examining these children, obesity was associated with height in 80% of cases against the background of elevated IGF-1 levels (25%) with normal growth hormone levels. The prevalence of high IGF-1 levels in children conceived with ART was also previously noted in a number of studies.^24^ When individually assessing carbohydrate disorders in obese children in this study, these disorders were revealed in the form of hypoglycemia (10%) and hypoinsulinemia (20%). There were no abnormalities in thyroid regulation in the thyroid gland of all obese children conceived after ART. However, half of the children were diagnosed with hypertriiodothyroninemia, which requires further research.

In children, physical development was also assessed using weight-to-height ratios. In this study, correct weight distribution in relation to height was observed in 85% of children born with ART and in 88.6% of NC children. Obesity was found at the same frequency of approximately 6% in both groups. In participants conceived using ART, a deficit in body weight relative to height was found in 9.2% of cases. It is important to clarify that almost every three children among them were tall. However, all of these parameters had no statistical significance depending on the conception option. The authors of this study evaluated the anthropometric parameters of the participants depending on the transfer of frozen or fresh embryos. In the sample of children born after FET, 75% of children were tall and 66.7% were overweight, which is consistent with previously obtained results on large baby syndrome born after FET.^30–32^

It is known that ART requires the use of hormone therapy. During the study, the parameters of sexual development in children were evaluated. Statistical differences in pathologies in both boys (cryptorchidism, hypoplasia) and girls (thelarche) were not found. In previous experiments, the association with urogenital defects in children after the ICSI method was demonstrated.^33–35^ However, in this study, no effect of the ICSI method on the development of genital pathology in boys was found (*p* = 0.758). When assessing thyroid function, most of the children examined were found to be euthyroid (median TSH and thyroxine levels were within the age reference values), which is consistent with the GUHS data.^22,36^ However, a statistically significant increase in free tbl3 levels was noted in the cohort of participants born after ART. In an individual assessment, there were increased tbl3 levels in 40% of cases in the group of children conceived with ART, compared with 30.5% of naturally born participants. This confirms the findings of Lv et al., in which a tendency towards an increase in tbl3 levels was observed in a group of children conceived with ART.^7^ Since this condition was found in both groups, it can be assumed that the increase in tbl3 levels in this study was related to the endemicity of iodine deficiency in the Republic of Kazakhstan. The prevalence of hypertriiodothyroninemia in the group of children conceived with ART requires further research.

When examining carbohydrate metabolism in children born with the help of ART, no abnormalities were found, and they did not differ from spontaneously conceived children, which confirms the conclusion of Guo et al.^14^ Glycemic indices in this study were within the reference values, but the median was significantly higher in the cohort of participants born with ART. During the regulation of carbohydrate metabolism in children of both groups, there was a statistically insignificant decrease in fasting insulinemia in about half of the participants in the sample. When comparing the indicators of insulin and glycemia, the frequency of hypoinsulinemic hypoglycemia was observed almost two times more often in the NC group. The results obtained require a more detailed assessment of nutrition, food quality, growth rates, and heredity in children, regardless of the method of conception. As is known, STH and its mediator IGF-1 are the regulators of growth rates in children. STH also has a counterinsular mechanism in glucose regulation. When assessing these hormones in participants born with ART, the median did not go beyond the reference and did not differ in the two comparison samples. A personal assessment of elevated IGF-1 levels revealed an almost identical ratio of cases in the group of children born with ART compared to NC children (19%:17.9%). In contrast to the control (2.5%:1.5%), tallness and elevated IGF-1 levels prevailed in the ART sample. Interestingly, all children with hypoinsulinemic hypoglycemia had elevated STH levels, with a predominance in the group of spontaneously conceived children (1:3).

The indicators of electrolytes in the blood (potassium and sodium) were determined to assess the mineralocorticoid function of the adrenal glands. A statistically significant increase in potassium levels was found in children conceived with ART, but the medians were within the reference values. An objective examination revealed no signs of adrenal insufficiency.

The inclusion of both NC and ART-conceived control groups, allowing direct comparison between the two populations, was a key strength of this work. Additionally, there was a relatively large sample size, which was determined using appropriate power estimates. Rather than just focusing on one or two analytes, the study covered a broad range of hormones related to development, metabolism, and endocrine function. The study design using clinical data sources was representative of actual clinical practice. Finally, objective anthropometric assessment methods and approved growth charts were used.

However, it is still important to take into account the limitations of this study. First, it was not possible to evaluate the long-term effects of ART on growth indices and hormone profiles later in life because the sample was limited to children under five years of age. Furthermore, only anthropometric measurements and circulating hormone levels were included in the data. No examination of the activities of hormones specific to certain tissues or a more thorough investigation of body composition was performed. Another potential drawback was the retrospective method of data collection from medical records, which would provide insufficient detail compared to a prospective study design. Finally, any confounding variables that may influence the assessed results, such as the mother's diet, level of exercise, or genetic background, were not taken into account.

Research on the impact of ART treatments on offspring's long-term health outcomes is ongoing. More current research findings are generally reassuring, although early investigations have raised concerns about possible increases in congenital malformations or epigenetic alterations. There are still unresolved concerns regarding the effect of ART on variables such as childhood growth patterns, obesity risk, glucose metabolism, and hormonal axes such as thyroid and growth hormone regulation. By providing a thorough investigation of several relevant hormones and anthropometrics in ART-conceived offspring compared to NC controls, this work strengthens the body of evidence. Understanding the subtle impacts of ART conception is crucial for early detection, patient counseling, and possibly even enhancing ART regimens.

## Conclusions

A comparative analysis of a number of biochemical and anthropometric indicators of children born after ART was conducted. Despite the use of hormone therapy in ART programs, the offspring did not show significant deviations in endocrine status. There was no statistically significant difference between the experimental (ART) and control (NC) groups in tbl4, ST, TSH, IGF, and insulin levels.

The method of reproductive technology also did not change the hormonal panel and biochemical parameters. Therefore, ART had no effect on the pubertal process. Both groups showed an increase in free tbl3 levels. Its titer was statistically significantly dependent on height. Insulin content depended on body weight and was reduced in overweight individuals. Body weight and IGF concentrations also correlated with height, glucose, and insulin levels by the type of direct weak association. There was a weak, inverse, statistically significant correlation between insulin titer and ST. NC children had large body weight and height at birth compared with children conceived with ART.

The prevalence of “large” children in the FET cohort is consistent with the literature data of other researchers. An increase in tbl3 levels in children conceived with ART requires further research to assess long-term health effects.

### List of abbreviations

[Table tbl16]

### Conflict of interest

The authors declare no conflict of interests.

### Authors’ contribution

Each author was involved in the concept and design, analysis and interpretation of the data, drafting and revising the manuscript, and approved the final manuscript. All authors agree to be accountable for all aspects of the work.

## Figures and Tables

**Table 1.** Number of studies conducted in comparison groups.

	Study groups	

Laboratory indicator	Main group (*n* = 120)	Control group (*n* = 132)

Free tbl4 level in the blood (mU/L)	119	131

Free tbl3 level in the blood (mU/L)	120	131

Blood TSH level (mU/L)	118	130

Insulin level in the blood (mU/ml)	119	131

IGF-1 level (ng/ml)	100	106

ST level (ng/ml)	100	106

Blood glucose level (mg/dl)	120	132

Blood potassium level (mmol/L)	120	132

Blood sodium level (mmol/L)	120	132


**Table 2.** Characteristics of the anamnestic data of mothers in the study groups.

	Study groups					

	Children after ART (*n* = 120)		NC children (*n* = 132)			OR; 95% confidence

Anamnestic data of mothers	Abs.*	%	Abs.	%	*p*	interval

Multiple births	22	18.3	3	2.3	< 0.001**	9.65; 2.81-33.18

Spontaneous miscarriage	12	10	19	14.4	0.339	0.661; 0.31-1.43

Non-developing pregnancies	13	10.8	27	20.5	0.037*	0.47; 0.23-0.97

Endometriosis	11	9.2	19	14.4	0.201	0.6; 0.27-1.32

Uterine fibroids	7	5.8	9	6.8	0.801	0.85; 0.31-2.35

Endometrial polyp	14	11.7	7	5.3	0.068	2.36; 0.92-6.06

Endometrial hyperplasia	6	5	2	1.5	0.156	3.42; 0.68-17.29

Ovarian cysts	12	10	18	13.6	0.373	0.7; 0.32-1.53

Arterial hypertension	5	4.2	4	3	0.74	1.39; 0.37-5.31

Type 1 and type 2 diabetes mellitus	0	0	2	1.5	0.325	0.22; 0.01-4.56

Thyroid disease	25	20.8	36	27.3	0.233	0.7; 0.39-1.26

Hypothyroidism	5	4.2	5	3.8	1	1.1; 0.31-3.91

Hyperthyroidism	0	0	2	1.5	0.325	0.22; 0.01-4.56

Hyperprolactinemia, prolactinomas	1	0.8	4	3	0.373	0.27; 0.03-2.44

Fatness	8	6.7	9	6.8	1	0.98; 0.36-2.62

Gestational diabetes	3	2.5	2	1.5	0.671	1.67; 0.27-10.15

Gestational thyrotoxicosis	1	0.8	0	0	0.463	3.33; 0.13-82.44


**The difference was significant (*p* < 0.05).

*Abs. – absolute number

**Table 3.** Comparison of anthropometric indicators in the study groups.

	Study groups				

Anthropometric	Children conceived by ART (*n* = 120)		NC children (*n* = 132)		

characteristics	Me [IQR]	Min-max	Me [IQR]	Min-max	*p*

Gestational age (weeks)	38 [36.3-39]	27-42	39 [38-40]	32-42	0.001*

Birth weight (g)	3,160 [2,695-3,600]	990-4,380	3,453 [3,140-3,690]	1,340-4,700	< 0.001*

Weight at the time of examination (g)	10,600 [8,500-12,500]	3,000-21,000	11,450 [10,000-14,000]	4,400-20,000	< 0.001*

Height at birth (cm)	51 [46.5-54]	35-58	53 [51-54]	38-60	0.002*

Height at the time of examination (cm)	79 [73-87]	51-107	87 [78-95]	60-108	< 0.001*


*The difference was significant (*p* < 0.05).

**Table 4.** Assessment of body weight by age in children in the study groups.

	Weight-to-age groups			

Study groups	From the 3rd to 97th percentile	>97 percentile	< 3 percentile	*p*

Children after ART (*n* = 120)	87.5% (105)	8.3% (10)	4.2% (5)	0.467

NC children (*n* = 132)	90.9% (120)	4.5% (6)	4.5% (6)	


**Table 5.** Assessment of age-related growth in the study groups.

	Age-related growth groups			

Study groups	From the 3rd to 97th percentile	>97 percentile	< 3 percentile	*p*

Children after ART (*n* = 120)	85% (102)	11.7% (14)	3.3% (4)	0.327

NC children (*n* = 132)	87.9% (116)	6.8% (9)	5.3% (7)	


**Table 6.** Assessment of weight to height in children in the study groups.

	Weight-to-height groups			

Study groups	From the 3rd to 97th percentile	>97 percentile	< 3 percentile	*p*

Children after ART (*n* = 120)	85% (102)	5.8% (7)	9.2% (11)	0.493

NC children (*n* = 132)	88.6% (117)	6.1% (8)	5.3% (7)	


**Table 7.** Assessment of the sexual development of boys in the study groups.

	Study groups				

Assessment of boys’	Boys after ART (*n* = 70)		NC boys (*n* = 71)		

sexual development	Abs.	%	Abs.	%	*p*

Norm	66	94.3	68	95.3	0.508

Unilateral cryptorchidism (*n* = 2)	2	2.9	2	2.8	

Bilateral cryptorchidism (*n* = 2)	0	0	1	1.4	

Penile hypoplasia (micropenis) (*n* = 2)	2	2.9	0	0	


**Table 8.** Results of comparing the incidence of genital abnormalities in boys conceived with ART (abs. (%)) depending on the method of conception.

	Study groups				

Assessment of boys’	Classical IVF (*n* = 29)		ICSI (*n* = 41)		

sexual development	Abs.	%	Abs.	%	*p*

Norm	28	96.6	38	92.7	0.758

Unilateral cryptorchidism (*n* = 2)	0	0	2	4.9	

Penile hypoplasia (micropenis) (*n* = 2)	1	3.4	1	2.4	


**Table 9.** Results of comparing the frequency of thelarche (abs. (%)) with different methods of conception.

	Study groups				

Assessment of the sexual	Girls after ART (*n* = 51)		NC girls (*n* = 57)		

development of girls	Abs.	%	Abs.	%	*p*

Norm	48	94.1	55	96.5	0.665

Thelarche	3	5.9	2	3.5	


**Table 10.** Parameters of the endocrine status of ART-conceived children compared to NC children.

	Study groups				

	Children after ART (*n* = 120)		NC children (*n* = 132)		

Parameters of endocrine status	Me [IQR]	Min-max	Me [IQR]	Min-max	*p*

Free tbl4 level in the blood (mU/L)	1.02 [0.92-1.08]	0.78-4.09	1.02 [0.94-1.09]	0.81-3.88	0.352

Free tbl3 level in the blood (mU/L)	3.65 [3.39-3.98]	2.69-4.69	3.48 [3.17-3.79]	1.04-4.59	0.002*

Blood TSH level (mU/L)	1.99 [1.53-3.01]	0.59-9.86	2.12 [1.59-2.83]	0.58-5.61	0.877

Insulin level in the blood (mU/ml)	2.8 [1.7-3.9]	0.7-17	2.5 [1.6-3.5]	0.3-16.2	0.212

IGF-1 level (ng/ml)	96.6 [66.5-151.2]	28.8-259.3	93.9 [68-137.7]	27.7-230.4	0.612

ST level (ng/ml)	2.65 [1.56-4.35]	0.28-18.9	2.36 [1.31-4.35]	0.34-17	0.356

Blood glucose level (mg/dl)	4.45 [4.2-4.7]	2.49-5.82	4.29 [3.96-4.62]	2.79-5.77	0.01

K^+^ concentration in the blood (mmol/L)	4.8 [4.5-5.1]	4-6.4	4.7 [4.4-4.9]	3.9-6.3	0.013

Na^+^ concentration in the blood (mmol/L)	139 [137-140]	121-151	139 [138-140.5]	134-149	0.064


*The difference was significant (*p* < 0.05).

**Table 11.** Endocrine status of children depending on the method of ART.

	Study groups				

	Classical IVF		ICSI		

Parameters of endocrine status	Me [IQR]	Min-max	Me [IQR]	Min-max	*p*

Level of free tbl4 in the blood (mU/L)	0.99 [0.91-1.06]	0.78-4.09	1.04 [0.95-1.09]	0.83-4.09	0.221

Level of free tbl3 in the blood (mU/L)	3.68 [3.42-4]	2.71-4.69	3.63 [3.38-3.93]	2.69-4.68	0.095

Blood TSH level (mU/L)	2.14 [1.68-2.95]	1.19-7.27	1.88 [1.44-2.95]	0.59-9.86	0.504

Insulin level in the blood (mU/ml)	2.9 [1.7-4.2]	0.7-7.6	2.7 [1.7-3.7]	0.9-17	0.699

IGF-1 level (ng/ml)	100.4 [72.7-159.1]	28.8-238.5	96 [66.2-144.3]	30.2-259.3	0.338

ST level (ng/ml)	2.06 [1.3-4.48]	0.4-18.9	2.8 [1.74-4.29]	0.28-10.2	0.443

Blood glucose level (mg/dl)	4.44 [4.22-4.71]	2.49-5.35	4.47 [4.19-4.76]	2.96-5.82	0.593

Concentration of K^+^ in the blood (mmol/L)	4.7 [4.5-5]	4-6.2	4.8 [4.5-5.1]	4-6.4	0.439

Na^+^ concentration in the blood (mmol/L)	139 [137-140]	121-151	138 [137-140]	134-145	0.363


**Table 12.** Endocrine status of children born after Fresh-ET or FET.

	Study groups				

	Fresh-ET		FET		

Parameters of endocrine status	Me [IQR]	Min-max	Me [IQR]	Min-max	*p*

Level of free tbl4 in the blood (mU/L)	1.01 [0.93-1.09]	0.81-1.19	1.03 [0.92-1.08]	0.78-4.09	0.865

Level of free tbl3 in the blood (mU/L)	3.62 [3.47-4.06]	3.1-4.68	3.66 [3.39-3.93]	2.69-4.69	0.755

Blood TSH level (mU/L)	1.76 [1.56-2.59]	0.99-4.69	2.07 [1.52-3.04]	0.59-9.86	0.334

Insulin level in the blood (mU/ml)	2.9 [1.8-5.5]	0.9-17	2.6 [1.6-3.7]	0.7-9.3	0.166

IGF-1 level (ng/ml)	81.6 [59.55-113.35]	31.7-158.6	102 [68.6-159]	28.8-259.3	0.058

ST level (ng/ml)	2.39 [1.58-3.78]	0.41-7.26	2.7 [1.55-4.56]	0.28-18.9	0.502

Blood glucose level (mg/dl)	4.45 [4.24-4.6]	4.03-5.82	4.45 [4.18-4.75]	2.49-5.35	0.607

Blood potassium level (mmol/L)	4.9 [4.6-5.4]	4-5.8	4.7 [4.5-5]	4-6.4	0.13

Blood sodium level (mmol/L)	139 [137-140]	136-143	139 [137-140]	121-151	0.785


**Table 13.** Percentage of detected deviations of the conducted studies in accordance with the reference values in the comparison groups.

	Study groups	

Laboratory indicator	Main group	Control group

Increased levels of free tbl4 in the blood	1.7% (2/119)	1.5% (2/131)

Elevated levels of free tbl3 in the blood	40% (48/120)	30.5% (40/131)

Elevated TSH levels in the blood	0.8% (1/118)	0.8% (1/130)

Reduced TSH levels in the blood	0.8% (1/118)	0.8% (1/130)

Reduced blood insulin levels	58% (69/119)	63.4% (83/131)

Elevated IGF-1 levels	19% (19/100)	17.9% (19/106)

Reduced IGF-1 levels	0% (0/100)	0.9% (1/106)

Increased ST level	7% (7/100)	13.2% (14/106)

Reduced blood glucose levels	10.8% (13/120)	19.7% (26/132)

Elevated blood potassium levels	22.5% (27/120)	12.9% (17/132)

Elevated blood sodium levels	1.7% (2/120)	2.3% (3/132)

Reduced blood sodium levels	6.7% (8/120)	7.6% (10/132)


**Table 14.** Results of the comparison of growth indicators (abs. (%)) depending on the detection of an increased level of free tbl3 in the blood of children conceived with ART.

	Height-for-age groups			

	3-97 percentile (*n* = 101)	>97 percentile (*n* = 14)	< 3 percentile (*n* = 4)	*p*

Elevated levels of free tbl3 in the blood	38 (37.6)	10 (71.4)	0 (0)	0.010* p_1-2_ = 0.016*

Reduced blood insulin levels	62 (61.4)	4 (28.6)	3 (75)	0.054


*The difference was significant (*p* < 0.05).

**Table 15.** Results of the comparison of the weight groups (abs. (%)) depending on the presence of reduced insulin levels in the blood of children conceived with ART.

	Weight-for-age groups			

	3-97 percentile (*n* = 104)	>97 percentile (*n* = 10)	< 3 percentile (*n* = 5)	*p*

Reduced blood insulin levels	62 (59.6)	2 (20)	5 (100)	0.006* p_1-2_ = 0.016*


*The difference was significant (*p* < 0.05).

**Table tbl16:** 

ART	Assisted Reproductive Technology

BMI	Body Mass Index

CI	Confidence Interval

FET	Frozen Embryo Transfer

GDM	Gestational Diabetes Mellitus

GUHS	Growing Up Healthy Study

HOMA-IR	Homeostasis Model Assessment of Insulin Resistance

ICSI	Intracytoplasmic Sperm Injection

IGF-1	Insulin-like Growth Factor 1

IVF	In Vitro Fertilization

K	Potassium

Na	Sodium

NC	Naturally Conceived

OR	Odds Ratio

PCOS	Polycystic Ovary Syndrome

ST	Somatotropin

STH	Somatotropic Hormone

T3	Triiodothyronine

T4	Total Thyroxine

TSH	Thyroid-Stimulating Hormone


## References

[bib1] Isenova SSh, Aripkhanova AS, Sultanmuratova DD, Kazybaeva AS, Tileukul NA, Boran AM (2023). Management strategies for thrombophilic patients undergoing assisted reproductive technologies. *Obstet Gynecol*.

[bib2] Opdahl S, Henningsen A-KA, Bergh C, Gissler M, Romundstad LB, Petzold M (2020). Data resource profile: Committee of Nordic Assisted Reproductive Technology and Safety (CoNARTaS) cohort. *Int J Epidemiol*.

[bib3] https://www.eshre.eu/europe/factsheets-and-infographics.

[bib4] (2021). *Hum Reprod Open*.

[bib5] Lokshin VN Practical reproductology. Almaty: KazMedPrint; 2023.

[bib6] Lokshin VN, Ilmuratova SK (2022). Cognitive development and neuropsychic health of children conceived by assisted reproductive technologies. *Obstet Gynecol*.

[bib7] Lv P-P, Meng Y, Lv M, Feng C, Liu Y, Li J-Y (2014). Altered thyroid hormone profile in offspring after exposure to high estradiol environment during the first trimester of pregnancy: a cross-sectional study. *BMC Med*.

[bib8] Coussa A, Hasan HA, Barber TM (2020). Impact of contraception and IVF hormones on metabolic, endocrine, and inflammatory status. *J Assist Reprod Genet*.

[bib9] Simbulan RK, Liu X, Feuer SK, Maltepe E, Donjacour A, Rinaudo P (2016). Adult male mice conceived by in vitro fertilization exhibit increased glucocorticoid receptor expression in fat tissue. *J Dev Orig Health Dis*.

[bib10] Mohammadi M, Morasae EK, Maroudizadeh S, Almasi-Hashiani A, Navid B, Amini P (2020). Assisted reproductive technology and the risk of gestational diabetes mellitus: a systematic review and meta-analysis. *Middle East Fertil Soc J*.

[bib11] Bosdou JK, Anagnostis P, Goulis DG, Lainas GT, Tarlatzis BC, Grimbizis GF (2020). Risk of gestational diabetes mellitus in women achieving singleton pregnancy spontaneously or after ART: a systematic review and meta-analysis. *Hum Reprod Update*.

[bib12] Dayan N, Fell DB, Guo Y, Wang H, Velez MP, Spitzer K (2018). Severe maternal morbidity in women with high BMI in IVF and unassisted singleton pregnancies. *Hum Reprod*.

[bib13] Fauser BCJM, Devroey P, Diedrich K, Balaban B, Bonduelle M, Delemarre-van de Waal HA (2014). Health outcomes of children born after IVF/ICSI: a review of current expert opinion and literature. *Reprod Biomed Online*.

[bib14] Guo X-Y, Liu X-M, Jin L, Wang T-T, Ullah K, Sheng J-Z (2017). Cardiovascular and metabolic profiles of offspring conceived by assisted reproductive technologies: a systematic review and meta-analysis. *Fertil Steril*.

[bib15] Zandstra H, van Montfoort APA, Dumoulin JCM, Zimmermann LJI, Touwslager RNM (2020). Increased blood pressure and impaired endothelial function after accelerated growth in IVF/ICSI children. *Hum Reprod Open*.

[bib16] Cui L, Zhou W, Xi B, Ma J, Hu J, Fang M (2020). Increased risk of metabolic dysfunction in children conceived by assisted reproductive technology. *Diabetologia*.

[bib17] Norrman E, Petzold M, Gissler M, Spangmose AL, Opdahl S, Henningsen A-K (2021). Cardiovascular disease, obesity, and type 2 diabetes in children born after assisted reproductive technology: a population-based cohort study. *PLoS Med*.

[bib18] Yeung EH, Mendola P, Sundaram R, Lin T-C, Broadney MM, Putnick DL (2022). Conception by fertility treatment and cardiometabolic risk in middle childhood. *Fertil Steril*.

[bib19] Steiner N, Wainstock T, Sheiner E, Walfisch A, Segal I, Haim A (2020). Long-term endocrine disorders in children born from pregnancies conceived following fertility treatments. *Early Hum Dev*.

[bib20] Belva F, Bonduelle M, Provyn S, Painter RC, Tournaye H, Roelants M (2018). Metabolic syndrome and its components in young adults conceived by ICSI. *Int J Endocrinol*.

[bib21] Juonala M, Lewis S, McLachlan R, Hammarberg K, Kennedy J, Saffery R (2020). American Heart Association ideal cardiovascular health score and subclinical atherosclerosis in 22-35-year-old adults conceived with and without assisted reproductive technologies. *Hum Reprod*.

[bib22] Penova-Veselinovic B, Wijs LA, Yovich JL, Burton P, Hart RJ (2022). Cohort profile: the Growing Up Healthy Study (GUHS) – a prospective and observational cohort study investigating the long-term health outcomes of offspring conceived after assisted reproductive technologies. *PLoS One*.

[bib24] Miles HL, Hofman PL, Peek J, Harris M, Wilson D, Robinson EM (2007). In vitro fertilization improves childhood growth and metabolism. *J Clin Endocrinol Metab*.

[bib25] Ueno K, Kojima J, Suzuki K, Kuwahara A, Higuchi Y, Tanaka A (2023). Anthropometric measurements of term singletons at 6 years of age born from fresh and frozen embryo transfer: A multicenter prospective study in Japan. *Reprod Med Biol*.

[bib26] Ono M, Kuji N, Ueno K, Kojima J, Nishi H (2024). The long-term outcome of children conceived through assisted reproductive technology. *Reprod Sci*.

[bib27] Laugesen K, Veres K, Hernandez-Diaz S, Chiu Y-H, Oberg AS, Hsu J (2023). Overweight or obesity in children born after assisted reproductive technologies in Denmark: a population-based cohort study. *PLoS Med*.

[bib28] Heslehurst N, Vieira R, Akhter Z, Bailey H, Slack E, Ngongalah L (2019). The association between maternal body mass index and child obesity: a systematic review and meta-analysis. *PLoS Med*.

[bib29] Ivashko M, Burmei S, Yusko L, Chaikovska T, Boyko N (2023). Microbiological diagnostics: from traditional to molecular genetic methods: a literature review. *Bull Med Biol Res*.

[bib30] Berntsen S, Pinborg A (2018). Large for gestational age and macrosomia in singletons born after frozen/thawed embryo transfer (FET) in assisted reproductive technology (ART). *Birth Defects Res*.

[bib31] Makhijani R, Bartels C, Godiwala P, Bartolucci A, Nulsen J, Grow D (2020). Maternal and perinatal outcomes in programmed versus natural vitrified-warmed blastocyst transfer cycles. *Reprod Biomed Online*.

[bib32] Ernstad EG, Wennerholm U-B, Khatibi A, Petzold M, Bergh C (2019). Neonatal and maternal outcome after frozen embryo transfer: Increased risks in programmed cycles. *Am J Obstet Gynecol*.

[bib33] Zhang Z, Liu X, Wei C, Luo J, Shi Y, Lin T (2021). Assisted reproductive technologies and the risk of congenital urogenital tract malformations: A systematic review and meta-analysis. *J Pediatr Urol*.

[bib34] Massaro PA, MacLellan DL, Anderson PA, Romao RLP (2015). Does intracytoplasmic sperm injection pose an increased risk of genitourinary congenital malformations in offspring compared to in vitro fertilization? A systematic review and meta-analysis. *J Urol*.

[bib35] Belva F, Bonduelle M, Roelants M, Michielsen D, Van Steirteghem A, Verheyen G (2016). Semen quality of young adult ICSI offspring: The first results. *Hum Reprod*.

[bib36] Komar TV, Khmara T-V, Tsyhykalo OV, Hrechko DI, Khmara AB (2023). Features of the blood supply of some areas of the head in human fetuses. *Bull Med Biol Res*.

